# Protein Kinase C Downregulation upon Rapamycin Treatment Attenuates Neuroinflammation and Mitochondrial Disease

**DOI:** 10.1093/geroni/igaa057.3281

**Published:** 2020-12-16

**Authors:** Anthony Grillo, Alessandro Bitto, Matt Kaeberlein

**Affiliations:** 1 University of Washington, Issaquah, Washington, United States; 2 University of Washington, Seattle, Washington, United States

## Abstract

Mitochondrial dysfunction causes many poorly understood diseases, such as Leigh Syndrome, that are often caused by dysfunctions in proteins involved in the electron transport chain. My lab previously reported mTOR is pathologically involved in the neurodegenerative phenotype and premature death of mice missing the Complex I subunit Ndufs4 (Ndufs4-/- mice). We discovered treatment with rapamycin extends lifespan, reduces neuroinflammation, and attenuates the neurodegenerative phenotype in these mice, although the mechanisms remain unclear. Rapamycin-treated Ndufs4-/- mice exhibited decreased activation of the mTORC1 pathway. It also deactivated the mTORC2 pathway. We observed that phosphorylation of the canonical protein kinase C (PKC) isoforms (PKC-α, -β, and -γ) decreased more than any other kinases, leading us to hypothesize its deactivation contributes to the observed lifespan extension. To test this, we treated Ndufs4-/- mice with three different PKC inhibitors: the pan-PKC inhibitors GO6983 and GF109203X, and the PKC-β specific inhibitor ruboxistaurin. Similar to rapamycin, all three drugs were able to significantly delay the onset of neurological symptoms (i.e. clasping) and increase survival. We also observed that PKC-β inhibition reduced skin inflammation to suppress the hair loss phenotype displayed by Ndufs4-/- mice at weaning. We further discovered PKC-β inhibition reduces neuroinflammation by deactivating the NF-kB inflammatory pathway. These results suggest that mTORC2 may play a critical role in the etiology of mitochondrial diseases such as Leigh Syndrome.

